# Trends and Rapidity of Dose Tapering Among Patients Prescribed Long-term Opioid Therapy, 2008-2017

**DOI:** 10.1001/jamanetworkopen.2019.16271

**Published:** 2019-11-15

**Authors:** Joshua J. Fenton, Alicia L. Agnoli, Guibo Xing, Lillian Hang, Aylin E. Altan, Daniel J. Tancredi, Anthony Jerant, Elizabeth Magnan

**Affiliations:** 1 Department of Family and Community Medicine, University of California, Davis, Sacramento; 2 Center for Healthcare Policy and Research, University of California, Davis, Sacramento; 3 OptumLabs, Minneapolis, Minnesota; 4 Department of Pediatrics, University of California, Davis, Sacramento

## Abstract

**Importance:**

A 2016 Centers for Disease Control and Prevention prescribing guideline cautioned against higher-dose long-term opioid therapy and recommended tapering daily opioid doses by approximately 10% per week if the risks outweigh the benefits. Warnings have since appeared regarding potential hazards of rapid opioid tapering.

**Objectives:**

To characterize US trends in opioid dose tapering among patients prescribed long-term opioids from 2008 to 2017 and identify patient-level variables associated with tapering and a more rapid rate of tapering.

**Design, Setting, and Participants:**

This retrospective cohort study used deidentified medical and pharmacy claims and enrollment records for 100 031 commercial insurance and Medicare Advantage enrollees, representing a diverse mixture of ages, races/ethnicities, and geographical regions across the United States. Adults with stable, higher-dosage (mean, ≥50 morphine milligram equivalents [MMEs]/d) opioid prescriptions for a 12-month baseline period and 2 or more months of follow-up from January 1, 2008, to December 31, 2017, were included in the study.

**Main Outcomes and Measures:**

Tapering was defined as 15% or more relative reduction in mean daily MME during any of 6 overlapping 60-day windows within a 7-month follow-up period. The rate of tapering was computed as the maximum monthly percentage dose reduction.

**Results:**

Among the 100 031 participants (53 452 [53.4%] women; mean [SD] age, 57.6 [11.8] years), from 2008 to 2015, the age- and sex-standardized percentage of patients tapering daily opioid doses increased from 10.5% to 13.7% (adjusted incidence rate ratio [aIRR] per year, 1.05 [95% CI, 1.05-1.06]) before increasing to 16.2% in 2016 and 22.4% in 2017 (aIRR in 2016-2017 vs 2008-2015, 1.20 [95% CI, 1.16-1.25]). Patient-level covariates associated with tapering included female sex (aIRR, 1.13 [95% CI, 1.10-1.15]) and higher baseline dose (aIRR for ≥300 MMEs/d vs 50-89 MMEs/d, 2.57 [95% CI, 2.48-2.65]). Among patients tapering daily opioid doses, the mean (SD) maximum dose reduction was 27.6% (17.0%) per month, and 18.8% of patients had a maximum tapering rate exceeding 40% per month (ie, faster than 10% per week). More rapid dose reduction was associated with 2016-2017 vs 2008-2015 (adjusted difference, 1.4% [95% CI, 0.8%-2.1%]) and higher baseline dose (adjusted difference, 2.7% [95% CI, 2.2%-3.3%] for 90-149 vs 50-89 MMEs/d).

**Conclusions and Relevance:**

Patients using long-term opioid therapy are increasingly undergoing dose tapering, particularly women and those prescribed higher doses; in addition, dose tapering has become more common since 2016. Many patients undergoing tapering reduce daily doses at a rapid maximum rate.

## Key Points

**Question:**

How often are patients who are prescribed long-term opioids undergoing tapering of their daily doses, and how often do patients undergo a rapid taper rate?

**Findings:**

This cohort study found that, among 100 031 patients with commercial or Medicare Advantage insurance who were using long-term opioids, the annual percentage undergoing tapering of their daily dosage increased from 10.5% in 2008 to 22.4% in 2017. Tapering was significantly more likely among women and patients with higher baseline opioid doses, and 18.8% of patents undergoing tapering had a maximum dose reduction rate exceeding 10% per week.

**Meaning:**

A substantial percentage of patients prescribed long-term opioid therapy are undergoing tapering, often at rapid maximum rates.

## Teaser

This cohort study characterizes US trends in opioid dose tapering among patients prescribed long-term opioids from 2008 to 2017 and identifies patient-level variables associated with tapering and a more rapid tapering rate.

## Introduction

Millions of individuals in the United States use opioids for chronic pain.^1^ A 2016 Centers for Disease Control and Prevention (CDC) prescribing guideline encouraged nonopioid therapies for chronic pain, questioned daily dosages above 50 morphine milligram equivalents (MMEs), and advised avoidance of daily dosages of 90 MMEs or higher.^2^ The guideline recommended tapering of opioids when the risks outweigh the benefits.^2,3,4^ The overall volume of opioid prescribing decreased contemporaneously with the publication of the guideline.^5^ Local or regional policies limiting opioid prescriptions or enforcing clinician use of prescription drug monitoring programs may also encourage opioid tapering.^6^

The CDC guideline recommended gradual dose reduction when tapering (ie, approximately 10% per week, possibly slower for patients with long-term use) to minimize withdrawal symptoms.^2^ This rate of dose reduction has been well tolerated by patients who are tapering opioid doses voluntarily while being closely monitored within multidisciplinary clinical programs,^7,8^ but there is limited evidence regarding optimal dose reduction rates in other clinical settings.^4^ In community practice, anecdotal reports suggest that many patients using long-term opioids are being forced to taper opioid doses involuntarily,^9^ and the US Food and Drug Administration (FDA) recently alerted health care professionals to avoid rapid opioid dose tapering among physically dependent patients because of potential hazards of serious withdrawal symptoms, uncontrolled pain, psychological distress, and suicide.^10,11^

To our knowledge, there are no systematic studies of the extent to which patients prescribed long-term opioid therapy undertake dose tapering or how frequently patients are rapidly tapering opioid doses. In this study, we examined trends in dose tapering among US patients prescribed long-term opioids, patient-level factors associated with dose tapering, and the rapidity of dose reduction among patients tapering long-term opioids. We hypothesized that dose tapering has become increasingly common in recent years, particularly after the publication of the 2016 CDC guideline,^2^ that higher baseline opioid doses would be associated with tapering, and that dose reduction would often occur at a pace that would be likely to precipitate adverse effects in long-term users.

## Methods

### Study Data, Setting, and Participants

This study used deidentified administrative claims data from the OptumLabs Data Warehouse (OLDW), which includes medical and pharmacy claims, laboratory test results, and enrollment records for commercial insurance and Medicare Advantage enrollees. The database contains longitudinal health information on enrollees and patients, representing a diverse mixture of ages, races/ethnicities, and geographical regions across the United States. The age and sex distributions of the OLDW commercial insurance and Medicare Advantage populations are similar to the distributions among the entire US commercial insurance and Medicare Advantage populations.^12^ This study used deidentified data in compliance with the Health Insurance Portability and Accountability Act Privacy Rule. Since the study involved the analysis of preexisting, deindentified data, it was exempt from Institutional Review Board approval by the University of California. This study followed the Strengthening the Reporting of Observational Studies in Epidemiology (STROBE) reporting guideline.

This study had a retrospective cohort design, using OLDW claims and enrollment data from January 1, 2008, to December 31, 2017, to identify a cohort of adult patients prescribed long-term, stable, higher doses of opioids based on the following criteria: (1) age 18 years or older on an opioid prescription fill date from January 1, 2008, to December 31, 2017; (2) at least 14 months of continuous commercial insurance or Medicare Advantage enrollment with medical, pharmacy, and mental health coverage, including both the opioid prescription fill date and the prior baseline year (360 days); (3) continuous oral or transcutaneous opioid therapy of 50 or more MMEs per day during the baseline year (operationalized as ≥90% proportion of days covered)^13^; (4) monthly mean opioid dose (in MMEs) that did not vary by more than 10% during any single month below or above the mean monthly dose across the entire baseline year (indicating dose stability); (5) absence of any nonskin cancer diagnosis during the baseline year (*International Classification of Diseases, Ninth Revision, Clinical Modification* codes 140-208, excluding 173, 184, and 187; *International Statistical Classification of Diseases and Related Health Problems, Tenth Revision* codes C00-C41 and C45-C96); (6) no evidence of hospice or palliative care and 90 days or fewer of nursing home, inpatient, skilled nursing, or long-term facility care during the baseline year; and (7) absence of any buprenorphine prescriptions during the baseline year (to exclude patients prescribed opioids as medication-assisted therapy for opioid use disorder).

We identified opioid prescription fills using National Drug Codes on pharmacy claims. Using the CDC Conversion Reference Table,^12^ we determined the daily opioid dose in MMEs based on the units dispensed and the number of days supplied, summing MMEs for all opioids prescribed on each date, including overlapping prescription fills.

Patients with a long-term and stable baseline opioid dose entered the study cohort and were considered at risk for opioid tapering events. The rationale for at least 14 months of continuous enrollment was to ensure that patients had a 12-month baseline period to establish a stable, long-term opioid dose and at least one 60-day postbaseline period for assessment of tapering. After the baseline period, patients were observed for tapering events during a follow-up period of up to 7 months unless censored owing to insurance disenrollment, a diagnosis of nonskin cancer, the use of palliative or hospice care, or death. The study design and analysis strategy allowed for multiple baseline and follow-up periods for patients, with potentially time-varying independent variables and variable follow-up durations.

### Identifying Opioid Tapering Events

To identify opioid tapering, we applied an empirically derived algorithm based on the moving mean daily dose within 6 overlapping 60-day windows during a 7-month follow-up period (eAppendix and eFigure 1 in the Supplement). We defined a tapering event as a 15% or greater reduction in mean daily opioid dose during any of the six 60-day windows during the 7-month follow-up period beginning at the end of the baseline period when compared with the mean dose during the baseline period. We computed moving mean daily doses within 60-day periods to smooth daily and monthly fluctuations arising from overlapping prescriptions or gaps between fills. The approach is sensitive enough to capture a 2.5% monthly dose reduction that accumulates to approximately 15% during a 6-month period (consistent with a very slow taper),^14^ but it also captures more abrupt dose reductions. For sensitivity analyses, we adapted the algorithm such that a tapering event was identified based on a 30% or more (rather than a ≥15%) relative reduction in mean daily opioid dose compared with the mean baseline dose.

### Estimating the Rapidity of Dose Tapering

Among patients who underwent tapering of opioid doses, we quantified the rapidity of dose tapering by determining the maximum rate of dose reduction (*V*) during the follow-up period, using the formula *V* = 100 × {1 − exp [ln (*T*/*B*)/*D*]}, where *T* and *B* are the tapered and baseline doses, *D* is the time in months from the most recent month at the baseline dose to the earliest month at the tapered dose, ln is the natural logarithm function, and exp is its inverse (see the eAppendix in the Supplement for detailed methods for identifying the maximum velocity of dose reduction). The formula yields the percentage dose reduction per month, with higher positive values indicating more rapid dose reduction. We assessed maximum, rather than mean, rate because patients would likely be at greatest risk for adverse effects during the period of maximum rate of dose reduction. We classified patients as having a maximum rate of more than 40% (vs ≤40%) per month because this threshold exceeds the maximum rate recommended by the CDC guideline (ie, approximately 10% per week, or 34.4% per month with 4 consecutive, weekly 10% reductions).^2^

### Covariates

Covariates included age (18-34, 35-50, 51-64, and ≥65 years), sex, race/ethnicity (white, black, Hispanic, Asian, or unknown), educational level (median educational level among residents ≥25 years within the patient’s census block group), insurance status (commercial vs Medicare Advantage), mean baseline opioid dose (50-89, 90-149, 150-299, or ≥300 MMEs), whether patients were coprescribed a benzodiazepine at any time during the baseline year (identified using National Drug Codes), rural vs urban residence (dichotomized as metropolitan or micropolitan vs small town or rural using Rural-Urban Commuting Area codes 1-6 vs 7-10),^15^ and a version of the Charlson Comorbidity Index (score: 0, 1, 2, or ≥3).^16^ We used an algorithm created by the CDC to determine whether patients had an emergency visit or hospitalization for drug overdose or toxic effects during the prior 90 days, hypothesizing that such events may prompt clinicians to initiate tapering of opioid doses.^17^ We specified time-varying covariates on the first date of each follow-up period. Data were missing or unknown for race/ethnicity for 2.6% of patients, for educational level for 5.1% of patients, and for rural vs urban residence for 0.1% of patients. For race/ethnicity and educational level, we created a missing or unknown indicator category, while we grouped the small number of patients with missing rural vs urban residence with the referent category (metropolitan or micropolitan).

### Statistical Analysis

We performed descriptive analyses to characterize the sample and to identify bivariate associations between patient characteristics, study year, and opioid tapering. To identify patient-level factors associated with tapering, we used Poisson regression for event history data to model tapering as a function of all independent variables, with the units of analysis being person-periods formed by segmenting each individual’s total follow-up time into nonoverlapping intervals.^18,19,20^ To account for potentially variable follow-up time within person-periods, all models included offsets. Because of an apparent stepwise increase in tapering beginning in 2016, we included a linear term for each year from 2008 to 2017 and a binary categorical variable for 2016 or 2017. After observing associations between age, sex, race/ethnicity, and baseline dose and tapering, we fit separate models that included interaction terms between each of these factors and the 2016-2017 categorical variable and used *z* tests and Akaike information criterion to assess whether interactions were informative.

To characterize age- and sex-standardized annual trends in opioid tapering overall and by baseline dose, we used Poisson regression to estimate adjusted incidence rate ratios (aIRRs) of tapering events associated, first, with study year, patient age, and sex, and, second, with study year, age, sex, baseline dose category and interactions between baseline dose category and year, and age category and year. We then used predictive margins to estimate the percentage of patients undergoing tapering by year, and by year and baseline dose.

We conducted descriptive analyses of maximum dose reduction rates among patients during tapering events. To identify patient-level factors associated with greater maximum rate of tapering, we used linear regression to model maximum rate as a function of the independent variables. To test the robustness of linear regression analyses of bounded rate data (ranging from 0 to 100), we used β regression to conduct a similar analysis of patient-level factors associated with higher maximum rate. We also used logistic regression to model the binary outcome of a maximum rate of more than 40% (vs ≤40%).

To account for clustering of follow-up periods within patients, we used cluster-robust SEs in all regression models. For analyses of factors associated with tapering, we obtained similar results using mixed-effects models with patient-level random effects. To test for potential bias stemming from missing data for race/ethnicity, educational level, or rural vs urban residence, we conducted complete case analyses for patient-level factors associated with tapering and maximum tapering rate. We defined statistical significance using an α of .05 with 2-tailed hypothesis tests. Analyses were conducted using Stata MP, version 15.1 (StataCorp).

## Results

The study sample included 100 031 patients prescribed long-term opioid therapy who had 174 822 periods of stable baseline dosing from 2008 to 2017 (mean [SD], 1.75 [1.19] per patient). More than half the sample (53 452 [53.4%]) were women, and the mean (SD) age was 57.6 (11.8) years (Table 1). A total of 55.8% of the sample had Medicare Advantage, including nearly one-third (32.3%) who were younger than 65 years and receiving Medicare owing to disability or end-stage renal disease; the remaining 44.2% patients had commercial insurance. Compared with patients with commercial insurance, patients with Medicare Advantage were more likely to be of black race/ethnicity, lower educational level, and higher comorbidity. Across the 174 822 stable baseline periods, 27 540 (15.8%) were followed by tapering events, with substantially higher percentages among patients receiving higher baseline opioid doses and in 2016 and 2017 (eTable 1 in the Supplement).

**Table 1.** Characteristics of Individual Patients Using Long-term Opioid Therapy by Insurance Coverage^a^

Characteristic	Patients, No. (%) (N = 100 031)	
	Commercial	Medicare Advantage
No. (%)	44 204 (44.2)	55 827 (55.8)
Age, y		
Mean (SD)	51.3 (10.1)	62.6 (10.7)
18-34	2721 (6.2)	301 (0.5)
35-49	15 011 (34.0)	5534 (9.9)
50-64	23 524 (53.2)	26 523 (47.5)
≥65	2948 (6.7)	23 469 (42.0)
Sex		
Male	21 434 (48.5)	25 145 (45.0)
Female	22 770 (51.5)	30 682 (55.0)
Race/ethnicity		
White	37 512 (84.9)	42 577 (76.3)
Black	2857 (6.5)	8289 (14.8)
Hispanic	2313 (5.2)	2912 (5.2)
Asian	365 (0.8)	412 (0.7)
Missing or unknown	1157 (2.6)	1637 (2.9)
Educational level^b^		
Less than high school	260 (0.6)	270 (0.5)
High school or equivalent	16 933 (38.3)	25 950 (46.5)
Some college	21 735 (49.2)	21 736 (38.9)
Bachelor degree or more	3688 (8.3)	1968 (3.5)
Missing or unknown	1588 (3.6)	5903 (10.6)
Urban vs rural residence		
Metropolitan	37 894 (85.7)	46 249 (82.8)
Micropolitan	3732 (8.4)	5359 (9.6)
Small town	1619 (3.7)	2748 (4.9)
Rural	835 (1.9)	1438 (2.6)
Missing or unknown	124 (0.3)	33 (0.1)
Charlson Comorbidity Index		
0	29 154 (66.0)	20 770 (37.2)
1	8997 (20.4)	15 642 (28.0)
2	3346 (7.6)	7892 (14.1)
≥3	2707 (6.1)	11 523 (20.6)
Baseline opioid dose, MMEs		
50-89	14 679 (33.2)	19 757 (35.4)
90-149	10 776 (24.4)	14 787 (26.5)
150-299	11 426 (25.8)	14 472 (25.9)
≥300	7323 (16.6)	6811 (12.2)
Coprescribed benzodiazepine	13 272 (30.0)	16 045 (28.7)
Recent drug overdose^c^	253 (0.6)	557 (1.0)

Abbreviation: MMEs, morphine milligram equivalents.

^a^ Patient characteristics as of the most recent eligible baseline period.

^b^ Median educational level of household members aged 25 years or older in the same residential zip code.

^c^ Recent drug overdose was defined as claims evidence of emergency department or inpatient visit for all-drug overdose within 90 days of the index opioid fill date.

The age- and sex-standardized percentage of patients tapering daily opioid doses increased from 10.5% in 2008 to 13.7% in 2015, before increasing to 16.2% in 2016 and 22.4% in 2017. In multivariate analyses with both linear (from 2008 to 2017) and step function (2016-2017) terms for study year, the following independent variables were statistically significantly associated with tapering: age 18 to 34 years (vs all other age groups), female sex (aIRR, 1.13 [95% CI, 1.10-1.15]), black race/ethnicity (vs white) (aIRR, 1.04 [95% CI, 1.01-1.08]), a Charlson Comorbidity Index score of 3 or higher (vs 0) (aIRR, 1.13 [95% CI, 1.09-1.17]), higher baseline dosage (aIRR for ≥300 MMEs/d vs 50-89 MMEs/d, 2.57 [95% CI, 2.48-2.65]), emergency department or hospital use for a drug overdose within the prior 90 days (aIRR, 1.36 [95% CI, 1.23-1.51]), coprescription of a benzodiazepine (aIRR, 1.02 [95% CI, 1.00-1.05]), and having commercial insurance (vs Medicare Advantage) (aIRR, 1.09 [95% CI, 1.06-1.12]) (Table 2). The model indicates that, in addition to a 5.4% linear annual increase in the adjusted rate of tapering from 2008 to 2017 (aIRR, 1.05 [95% CI, 1.05-1.06]), the adjusted rate of tapering increased by 20.4% in 2016-2017 (IRR, 1.20 [95% CI, 1.16-1.25]). Similar patient-level factors were associated with tapering when the algorithm was adapted to use a 30% or more (rather than a ≥15%) dose reduction threshold (eTable 2 in the Supplement) and in a complete case analysis (eTable 3 in the Supplement). When analyses were restricted to follow-up periods from 2015 to 2017, the associations between baseline dose and tapering were stronger, and the association between age and coprescription of a benzodiazepine and tapering were no longer statistically significant (eTable 4 in the Supplement).

**Table 2.** Poisson Regression Analysis of Patient-Level Factors Associated With Long-term Opioid Dose Tapering^a^

Independent Variable	aIRR (95% CI)	*P* Value
Age, y		
18-34	1 [Reference]	NA
35-49	0.89 (0.83-0.95)	.001
50-64	0.84 (0.79-0.90)	<.001
≥65	0.87 (0.81-0.93)	<.001
Sex		
Male	1 [Reference]	NA
Female	1.13 (1.10-1.15)	<.001
Race/ethnicity		
White	1 [Reference]	NA
Black	1.04 (1.01-1.08)	.02
Hispanic	1.01 (0.96-1.06)	.73
Asian	0.93 (0.82-1.07)	.31
Missing or unknown	0.97 (0.90-1.04)	.40
Educational level^b^		
High school or less	1 [Reference]	NA
More than high school	0.99 (0.96-1.01)	.20
Missing or unknown	0.99 (0.95-1.04)	.79
Urban vs rural residence		
Metropolitan, micropolitan, or unknown	1 [Reference]	NA
Small town or rural	1.00 (0.95-1.04)	.87
Charlson Comorbidity Index		
0	1 [Reference]	NA
1	1.04 (1.01-1.07)	.009
2	1.03 (0.99-1.06)	.18
≥3	1.13 (1.09-1.17)	<.001
Baseline opioid dose, MMEs		
50-89	1 [Reference]	NA
90-149	1.49 (1.44-1.54)	<.001
150-299	1.94 (1.88-2.00)	<.001
≥300	2.57 (2.48-2.65)	<.001
Coprescribed benzodiazepine	1.02 (1.00-1.05)	.048
Recent drug overdose^c^	1.36 (1.23-1.51)	<.001
Insurance		
Medicare Advantage	1 [Reference]	NA
Commercial	1.09 (1.06-1.12)	<.001
Year^d^		
Linear term per year (2008-2017)	1.05 (1.05-1.06)	<.001
2016-2017	1.20 (1.16-1.25)	<.001

Abbreviations: aIRR, adjusted incidence rate ratio; MMEs, morphine milligram equivalents; NA, not applicable.

^a^ There were 174 822 stable periods of opioid use among 100 031 patients.

^b^ Median educational level of household members aged 25 years or older in the same residential zip code.

^c^ Recent drug overdose was defined as claims evidence of emergency department or inpatient visit for all-drug overdose within 90 days of the index opioid fill date.

^d^ Study year was modeled by including a linear term for each increase in study year from 2008 to 2017 and an indicator variable for the 2016-2017 period.

The Figure demonstrates the gradual increase in age- and sex-standardized percentages of patients undergoing opioid dose tapering from 2008 to 2015 followed by a more marked increase in 2016 and 2017 (Figure, A). We found a statistically significant interaction between baseline opioid dose and the size of the stepped increase in tapering in 2016 and 2017. The age- and sex-standardized percentages of patients undergoing tapering was higher in all years from 2008 to 2017 among patients with higher baseline doses compared with patients with baseline doses of 50 to 89 MMEs, and the percentage of patients undergoing tapering increased more substantially in 2016 and 2017 among patients in the higher baseline dose groups (Figure, B). We estimate that, in 2017, 29.2% (95% CI, 28.1%-30.3%) of patients prescribed baseline doses of 150 to 299 MMEs and 43.5% (95% CI, 41.4%-45.6%) of patients prescribed baseline doses of 300 MMEs or higher underwent tapering. Similar secular trends were observed using a 30% or more dose reduction threshold to identify tapering (eFigure 2 in the Supplement).

**Figure.**
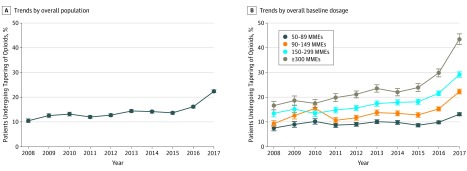
Age- and Sex-Standardized Rates of Dose Tapering Among Patients Using Long-term Opioid Therapy, 2008-2017 A, Trends among the overall population. B, Trends by baseline dosage in morphine milligram equivalents (MMEs). Error bars indicate 95% CIs.

During the 27 540 tapering events, the mean (SD) maximum rate of dose tapering was 27.6% (17.0%) per month. Consistent with skewing toward faster maximum rates, the median maximum rate was 22.3% with a 5th percentile of 9.8%, a 25th percentile of 15.6%, a 75th percentile of 34.3%, and a 95th percentile of 64.9%. Nearly one-fifth of patients (18.8%) had a maximum rate exceeding 40% per month. In multiple linear regression analysis, the following patient-level variables were statistically significantly associated with a higher maximum dose reduction rate: a high school education or less (vs more education), a Charlson Comorbidity Index score of 3 or higher (vs 0) (adjusted difference, 1.8% [95% CI, 1.1%-2.4%]), higher baseline doses (vs 50-89 MMEs) (adjusted difference, 2.7% [95% CI, 2.2%-3.3%] for 90-149 vs 50-89 MMEs), a recent drug overdose (adjusted difference, 4.5% [95% CI, 2.2%-6.8%]), and the 2016-2017 period (vs earlier years) (adjusted difference, 1.4% [95% CI, 0.8%-2.1%]) (Table 3). These variables were similarly associated with a higher maximum tapering rate when analyzed using β regression (eTable 5 in the Supplement), in a complete case analysis (eTable 6 in the Supplement), and in a logistic regression analysis of a maximum rate exceeding 40% per month (eTable 7 in the Supplement).

**Table 3.** Linear Regression Analysis of Patient-Level Factors Associated With Maximum Month-to-Month Dose Reduction Rates Among Patients Tapering Opioid Dose^a^

Characteristic	β (95% CI)^b^	*P* Value
Age, y		
18-34	[Reference]	NA
35-49	–0.7 (–2.0 to 0.7)	.33
50-64	–1.1 (–2.4 to 0.2)	.10
≥65	–0.5 (–1.9 to 0.9)	.50
Sex		
Male	[Reference]	NA
Female	0.2 (–0.2 to 0.6)	.77
Race/ethnicity		
White	[Reference]	NA
Black	0.6 (0.0 to 1.3)	.06
Hispanic	–0.2 (–1.1 to 0.7)	.70
Asian	–0.7 (–3.0 to 1.5)	.53
Missing or unknown	0.3 (–1.0 to 1.6)	.66
Educational level^c^		
High school or less	[Reference]	NA
More than high school	–1.1 (–1.5 to –0.7)	<.001
Missing, unknown, or missing	0.1 (–0.8 to 1.1)	.81
Urban vs rural residence		
Metropolitan, micropolitan, or unknown	[Reference]	NA
Small town or rural	0.4 (–0.4 to 1.3)	.33
Charlson Comorbidity Index		
0	[Reference]	NA
1	0.0 (–0.5 to 0.5)	.89
2	0.6 (0.0 to 1.3)	.07
≥3	1.8 (1.1 to 2.4)	<.001
Baseline opioid dose, MMEs		
50-89	[Reference]	NA
90-149	2.7 (2.2 to 3.3)	<.001
150-299	3.0 (2.4 to 3.5)	<.001
≥300	2.6 (2.0 to 3.2)	<.001
Coprescribed benzodiazepine	0.3 (–0.1 to 0.8)	.13
Recent drug overdose^d^	4.5 (2.2 to 6.8)	<.001
Insurance		
Medicare Advantage	[Reference]	NA
Commercial	–0.2 (–0.7 to 0.3)	.37
Year^e^		
Linear term per year (2008-2017)	–0.4 (–0.5 to –0.3)	<.001
2016-2017	1.4 (0.8 to 2.1)	<.001
Constant	25.2 (23.7 to 26.7)	<.001

Abbreviations: MME, morphine milligram equivalents; NA, not applicable.

^a^ There were 27 540 dose tapering events among 25 471 patients.

^b^ The β coefficients estimate the percentage change maximum dose reduction rate associated with covariates. Positive estimates indicate a more rapid maximum rate of dose reduction.

^c^ Median educational level of household members aged 25 years or older in the same residential zip code.

^d^ Recent drug overdose was defined as claims evidence of emergency department or inpatient visit for all-drug overdose within 90 days of the index opioid fill date.

^e^ Study year was modeled by including a linear term for each increase in study year from 2008 to 2017 and an indicator variable for the 2016-2017 period.

## Discussion

Among a cohort of US patients with commercial insurance and Medicare Advantage, the percentage prescribed long-term opioids undergoing dose tapering has increased substantially in recent years, particularly after the publication of the 2016 CDC opioid prescribing guideline^2^ and among patients prescribed daily doses exceeding 90 MMEs. The CDC guideline authors recently addressed the misapplication of guideline elements,^11^ and particularly cautions regarding daily dosages above 90 MMEs, which some clinicians and pharmacies may have interpreted as a “hard stop,” prompting dose tapering among many patients receiving long-term, stable doses of 90 MMEs or higher.^21^ An expert panel and the CDC authors have emphasized that clinicians caring for patients receiving long-term therapy with opioids should routinely reassess the benefits and risks of ongoing opioid therapy and reduce opioid doses only if the risks seem to outweigh the benefits.^11,21^ Experts have also advised that dose reduction should be considered collaboratively with patients and be initiated with the patients’ consent whenever possible, avoiding forced or mandatory tapers, which may pose a greater risk of iatrogenic harm than tapers initiated voluntarily.^9,21^

After reports of patients experiencing withdrawal symptoms, psychological distress, and suicidality after rapid dose tapering, the FDA recently issued a safety announcement cautioning clinicians regarding the potential hazards of rapid dose reduction among patients taking long-term opioids.^10^ In 2016, the CDC guideline authors advised that a dose reduction of 10% per week is “a reasonable starting point”^2^^(p1639)^ when tapering and cautioned that tapers as slow as 10% per month may be better tolerated among patients taking long-term opioids.^3^ These recommendations imply that monthly dose reductions of 10% to 40% may be clinically reasonable. In our cohort, nearly 1 in 5 patients tapered at a rate faster than 40% per month, and 5% of patients tapered at a rate faster than 60% per month.

The rate of fatal opioid overdose among men is twice the rate among women,^22,23^ and men are more likely than women to misuse opioids.^24^ However, the women in our sample had a 13% higher aIRR of dose tapering than men. Female sex was also associated with opioid tapering among patients prescribed long-term opioids within a single academic health system.^25^ Few studies have evaluated sex differences in pain-related behaviors that may be associated with physician prescribing.^26,27,28^ Although women in a pain clinic setting were more likely than men to stockpile opioid medication,^26^ men exhibited higher rates of concurrent alcohol or illicit drug abuse or unauthorized dose increases.^26,28^ When considering dose tapering for patients, clinicians may fear that a recommendation of tapering may prompt angry or even violent responses, particularly from male patients.^29,30^ Such perceptions may be associated with a sex bias among clinicians, manifesting as a greater willingness to initiate tapering among women than men.

Prior studies have documented disparities in pain management between black and white patients,^31,32^ including more frequent urinary drug monitoring among black patients with long-term use of opioids,^33^ higher rates of opioid discontinuation when urine test results reveal illicit drugs,^34^ and, within a single academic health system, higher rates of tapering long-term opioids.^25^ In adjusted analyses, black patients were slightly more likely than white patients to undertake opioid tapering. In light of potential residual confounding, the small magnitude of this difference is of uncertain clinical significance.

Coprescription of opioids and benzodiazepines is associated with increased risk of opioid overdose,^35,36,37^ and the CDC guideline recommends avoidance of sedative coprescription for patients taking long-term opioids.^2^ Despite these recommendations, we found only a weak association between benzodiazepine coprescription and opioid tapering that disappeared in analyses restricted to 2015-2017. Our study, however, did not distinguish patients based on benzodiazepine dose or duration of use during baseline years.

Overdose events are associated with substantial risk for subsequent overdose,^38^ so the higher rate of tapering among patients with recent emergency department or hospital visits for overdose seems clinically appropriate. Nevertheless, as shown in eTable 1 in the Supplement, less than one-fourth of patients with recent overdoses underwent dose tapering (23.4%), consistent with other evidence that patients frequently receive ongoing opioid prescriptions after opioid overdose.^38,39^

### Limitations

This study has some limitations. The data source captured data only from US patients with commercial insurance or Medicare Advantage; the generalizability of our findings to uninsured, Medicaid, or non-US populations is uncertain. Although we could not validate our tapering measure against a criterion standard, our findings support the convergent validity of the measure, as one would expect both a secular increase in tapering and a correlation between baseline opioid dose and tapering. Nevertheless, study data are derived from administrative claims, and measurement error may have occurred. In addition, pharmacy claims records do not capture self-paid prescriptions or opioids dispensed within methadone maintenance programs. Although our tapering measure identifies dose reductions, it does not evaluate the sustenance of these dose reductions; indeed, many tapering events may not have been sustained. We also could not ascertain whether tapering was initiated by prescribers or patients.

## Conclusions

Opioid tapering has become increasingly common among patients using long-term opioids, particularly among patients taking higher doses and since the publication of the CDC opioid prescribing guideline.^2^ Our results also suggest that many patients undergo tapering at rapid maximum rates. The downstream effects of opioid tapering on pain, withdrawal, mental health, and overdose risk warrant careful evaluation.

## Notes

Supplement. **eAppendix.** Methods **eFigure 1.** Algorithm for Identifying Tapering Events Based on 60-Day Moving Average Dose During 7-Month Postbaseline Period **eTable 1.** Baseline Periods of Stable Opioid Use and Subsequent Tapering Events by Patient Characteristics and Year **eTable 2.** Poisson Regression Analyses Assessing Patient-Level Predictors of Tapering Based on a ≥30% Relative Dose Reduction Threshold (N=174,822 Stable Periods of Opioid Use Among 100,031 Patients) **eTable 3.** Complete Case Poisson Regression Analysis of Patient-Level Predictors of Long-term Opioid Dose Tapering (N=162,345 Stable Periods of Opioid Use Among 90,613 Patients) **eTable 4.** Poisson Regression Analyses Assessing Patient-Level Predictors of Tapering, Restricted to Follow-up Periods in 2015-2017 (N=77,103 Stable Periods of Opioid Use Among 54,761 Patients) **eFigure 2.** Age- and Sex-Standardized Rates of Tapering Based on a ≥30% Relative Dose Reduction Threshold Among Patients Using Long-Term Opioid Therapy, 2008-2017 **eTable 5.** Beta Regression Analysis Assessing Patient-Level Predictors of Higher Maximum Tapering Velocity (N=27,540 Tapering Events Among 25,471 Patients) **eTable 6.** Complete Case Linear Regression Analysis Assessing Patient-Level Predictors of Maximum Tapering Velocity (N=25,401 Tapering Events Among 23,400 Patients) **eTable 7.** Logistic Regression Analysis of Patient-Level Predictors of Maximum Tapering Velocity Exceeding 40% per Month (N=27,540 Tapering Events Among 25,471 Patients) **eReference**
