# Data Processing and Patient Eligibility Indicator Errors in Study About Dose Tapering Among Patients Prescribed Long-term Opioid Therapy

**DOI:** 10.1001/jamanetworkopen.2020.18893

**Published:** 2020-08-17

In the Original Investigation, “Trends and Rapidity of Dose Tapering Among Patients Prescribed Long-term Opioid Therapy, 2008-2017,”^1^ that was published in *JAMA Network Open* on November 15, 2019, a data processing error occurred that resulted in an underestimation of the overall frequency of tapering and how rapidly patients are having their doses reduced. In addition, OptumLabs informed the authors that an indicator of patient eligibility for pharmacy benefits was incorrect for a small number of patients during the study period, which resulted in a corrected overall sample size of 99 874 participants (vs the previously reported 100 031 participants). The overall frequency of dose tapering in the original publication was underreported as 10.5% in 2008 and 22.4% in 2017; the corrected estimates are 12.7% in 2008 and 23.1% in 2017. The correct number of tapering events is 30 255 (vs 27 540), and the analysis includes 4129 tapering events in which the tapered dose was to 0 opioids (13.7% of all tapering events). Because of changes to the sample and the identification of more tapering events, the incidence rate ratios for patient-level variables associated with tapering reported in Table 2 have been corrected. The mean maximum rate of dose reduction is greater than previously estimated (34.0% per month vs 27.6% per month) and has greater skew toward very rapid dose reduction, with 26.5% of patients tapered at a maximum rate of 40% or greater per month. The data in Table 3 have also been corrected. In the corrected article, a higher maximum dose reduction rate was associated with an age of 18 to 34 years (vs older age groups), male sex, a high school education or less (vs more education), small town or rural residence (vs metropolitan, micropolitan, or unknown), a Charlson Comorbidity Index score of 3 or higher (vs 0), lower baseline doses (50-89 morphine milligram equivalents vs 150-299 or ≥300 morphine milligram equivalents), and a recent drug overdose. A new eTable 8 has been added to show the percentage of tapering episodes with dose reductions to 0 opioids. Years 2016 and 2017 were no longer statistically significantly associated with increased rate of dose reduction in corrected analyses. To address these errors, corrections have been made to the Abstract; Methods; Results; Discussion; Tables 1, 2, and 3; the Figure; and the Supplement. The article has been corrected online,^1^ and the authors have published an explanation as a Comment^2^ linked to the article.
