# Supplementary material for: Trends and Rapidity of Dose Tapering Among Patients Prescribed Long-term Opioid Therapy, 2008-2017
Source: JAMA Netw Open. 2019 Nov 15;2(11):e1916271. doi: 10.1001/jamanetworkopen.2019.16271 (PMC6902834; doi:10.1001/jamanetworkopen.2019.16271)
Supplement: Supplement. — eAppendix. Methods eFigure 1. Algorithm for Identifying Tapering Events Based on 60-Day Moving Average Dose During 7-Month Postbaseline Period eTable 1. Baseline Periods of Stable Opioid Use and Subsequent Tapering Events by Patient Characteristics and Year eTable 2. Poisson Regression Analyses Assessing Patient-Level Predictors of Tapering Based on a ≥30% Relative Dose Reduction Threshold (N=174,822 Stable Periods of Opioid Use Among 100,031 Patients) eTable 3. Complete Case Poisson Regression Analysis of Patient-Level Predictors of Long-term Opioid Dose Tapering (N=162,345 Stable Periods of Opioid Use Among 90,613 Patients) eTable 4. Poisson Regression Analyses Assessing Patient-Level Predictors of Tapering, Restricted to Follow-up Periods in 2015-2017 (N=77,103 Stable Periods of Opioid Use Among 54,761 Patients) eFigure 2. Age- and Sex-Standardized Rates of Tapering Based on a ≥30% Relative Dose Reduction Threshold Among Patients Using Long-Term Opioid Therapy, 2008-2017 eTable 5. Beta Regression Analysis Assessing Patient-Level Predictors of Higher Maximum Tapering Velocity (N=27,540 Tapering Events Among 25,471 Patients) eTable 6. Complete Case Linear Regression Analysis Assessing Patient-Level Predictors of Maximum Tapering Velocity (N=25,401 Tapering Events Among 23,400 Patients) eTable 7. Logistic Regression Analysis of Patient-Level Predictors of Maximum Tapering Velocity Exceeding 40% per Month (N=27,540 Tapering Events Among 25,471 Patients) eReference [file jamanetwopen-2-e1916271-s001.pdf]

Supplementary Online Content

Fenton JJ, Agnoli AL, Xing G, et al. Trends and rapidity of dose tapering among patients prescribed long-term opioid therapy, 2008-2017. *JAMA Netw Open.* 2019;2(11):e1916271. doi:10.1001/jamanetworkopen.2019.16271

eAppendix. Methods
eFigure 1. Algorithm for Identifying Tapering Events Based on 60-Day Moving Average Dose During 7-Month Postbaseline Period
eTable 1. Baseline Periods of Stable Opioid Use and Subsequent Tapering Events by Patient Characteristics and Year
eTable 2. Poisson Regression Analyses Assessing Patient-Level Predictors of Tapering Based on a ≥30% Relative Dose Reduction Threshold (N=174,822 Stable Periods of Opioid Use Among 100,031 Patients)
eTable 3. Complete Case Poisson Regression Analysis of Patient-Level Predictors of Long-term Opioid Dose Tapering (N=162,345 Stable Periods of Opioid Use Among 90,613 Patients)
eTable 4. Poisson Regression Analyses Assessing Patient-Level Predictors of Tapering, Restricted to Follow-up Periods in 2015-2017 (N=77,103 Stable Periods of Opioid Use Among 54,761 Patients)
eFigure 2. Age- and Sex-Standardized Rates of Tapering Based on a ≥30% Relative Dose Reduction Threshold Among Patients Using Long-Term Opioid Therapy, 2008-2017
eTable 5. Beta Regression Analysis Assessing Patient-Level Predictors of Higher Maximum Tapering Velocity (N=27,540 Tapering Events Among 25,471 Patients)
eTable 6. Complete Case Linear Regression Analysis Assessing Patient-Level Predictors of Maximum Tapering Velocity (N=25,401 Tapering Events Among 23,400 Patients)
eTable 7. Logistic Regression Analysis of Patient-Level Predictors of Maximum Tapering Velocity Exceeding 40% per Month (N=27,540 Tapering Events Among 25,471 Patients)
eReference

This supplementary material has been provided by the authors to give readers additional information about their work.

© 2019 Fenton JJ et al. *JAMA Network Open.*

eAppendix. Methods

Algorithm for Identifying Tapering Events

During descriptive and graphical analyses of dosing patterns, we found substantial day-to-day fluctuation in dosing attributable to overlapping prescriptions or gaps in fills. To smooth short-term fluctuations in average daily dose, we opted to compute a moving average of the daily dose within 60-day follow-up periods following the baseline period of stable dosage. As shown in eFigure 1 below**,** the approach uses recursive logic to compare the average baseline opioid dose to average daily opioid doses across six overlapping 60-day windows during a 7 month follow-up period. For primary analyses, we define a tapering event as a ≥15% relative reduction in average daily opioid dose during any of six 60-day windows during the seven-month follow-up period beginning at the end of the 12-month baseline period of stable dosing. The comparisons of baseline and follow-up average doses are sequential, beginning with the first 60-day average post-baseline (F1 and proceeding to the second through the sixth 60-day period (F2-F6), assessing at each point whether a ≥15% reduction in average daily dose is present relative to the stable baseline dose. The approach is sensitive enough to capture a 2.5% monthly dose reduction that accumulates to ~15% over a six-month period (consistent with a "very slow taper" per Veterans Affairs guidelines),[1] but also captures more abrupt dose reductions. For sensitivity analyses, we analogously defined a tapering event as a ≥30% relative reduction in average daily opioid dose during any of six 60-day windows during the seven-month follow-up period beginning at the end of the 12-month baseline period of stable dosing.

If at any point during the follow-up period, a patient's average daily dose during a 60-day period increased by 10% above the stable baseline dose, we classified the patient as having an unstable dose and not tapered during the follow-up. Indeed, we observed that patients with large percentage increases in dispensed MME during a single 60-day follow-up period often had compensatory drops in dispensed MME during the subsequent 60-day period. Such apparent dose increases could arise when patients accumulate a stockpile of doses during months of vacation or travel, etc. Hence, the subsequent drop in dispensed MME would be expected and would not necessarily represent a tapering event. Because we classified these patients as having unstable doses based on the increase of their monthly dose greater than 10% above the stable baseline dose, our approach did not classify such a pattern as tapering.

Determining the Maximum Velocity of Dose Tapering

Among patients with tapering events, we used the following formula to determine the velocity of dose reduction (V) during the follow-up period:

$$V = 100 * [1 - \exp [\ln(T/B) / D]],$$

where T and B are the tapered and baseline doses, D is the time in months from the most recent month at the baseline dose to the earliest month at the tapered dose, ln() is the natural logarithm function, and exp() is its inverse. The formula computes the monthly percent dose reduction with higher values indicating more rapid dose reduction and a maximum of 100, signifying a 100% dose reduction from one study month to the next.

For patients classified as tapering, we used a four-step process to identify the maximum velocity. The first step was to compute an array of up to 21 velocities based on comparisons of all possible baseline and tapering doses across the seven-month follow-up period, beginning with the six follow-up periods (F1-F6) relative to the baseline period (B) and continuing with the five latest follow-up periods (F2-F6) relative to the first follow-up period (F1), etc. (For patients with shorter follow-up periods due to censoring, the array of velocities was correspondingly smaller.)

The second step was to exclude all velocities that were derived from follow-up periods that occurred after any 60-day period during which the average daily dose increased by 10% or more above the baseline dose. (As above, we classified these patients as having an "unstable dose" during these periods.) We excluded velocity calculations based on months after dose instability because we observed very fast velocities in instances when the dispensed MME during a 60-day periods dropped precipitously after a transient increase in average daily dose above the baseline line dose. After excluding velocities after periods of dose instability, the remaining velocities all derived from period-to-period calculations during the period of dose reduction among tapering patients.

The third step was to exclude all velocity measurements after 60-day periods when the average daily dose was ≤50 MME. The rationale for this exclusion is that small absolute changes in daily doses below 50 MME may be associated with large percentage changes in month-to-month dose reduction.

In the fourth and final step, we selected the maximum velocity from the remaining velocities. This measure captures the maximum month-to-month percentage change in average daily dose among tapering patients during the tapering period and when the average daily dose was ≥50 MME.

© 2019 Fenton JJ et al. *JAMA Network Open.*

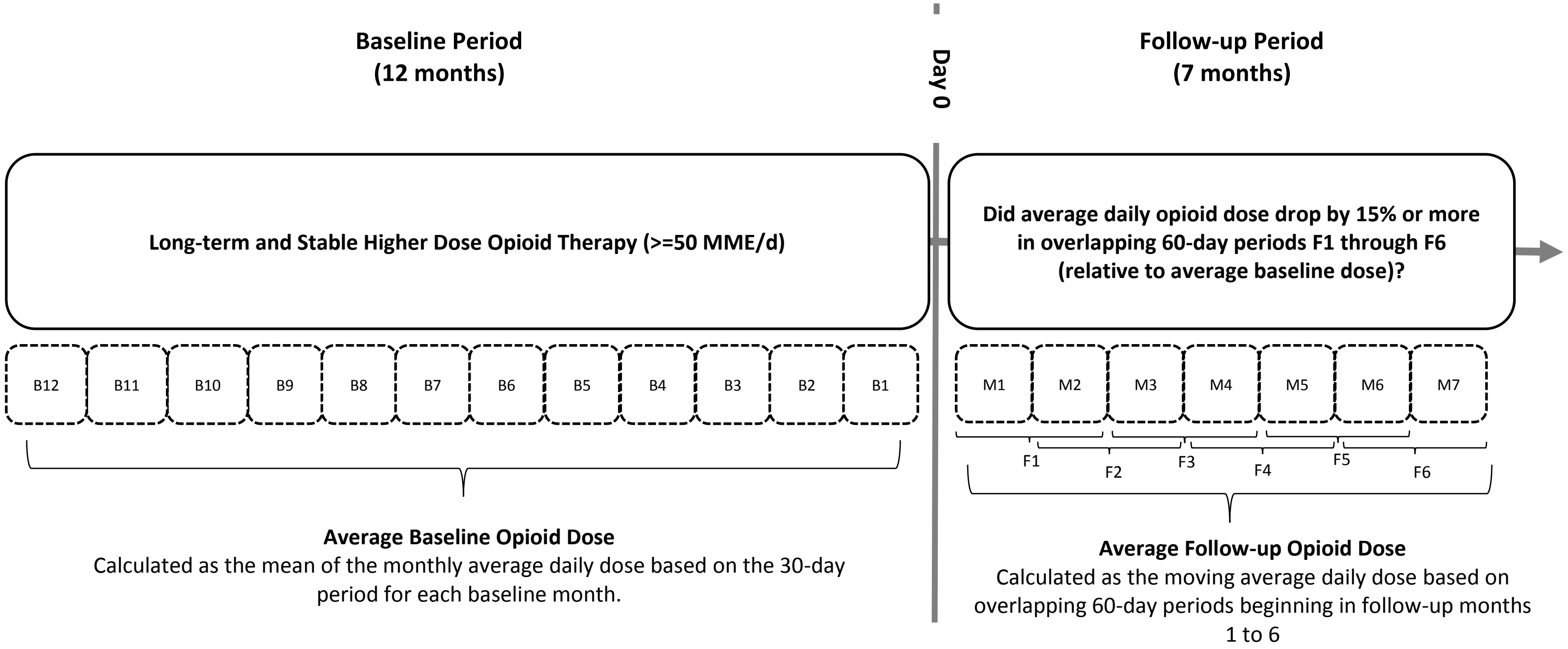

eFigure 1. Algorithm for Identifying Tapering Events Based on 60-Day Moving Average Dose During 7-month Postbaseline Period
B1-B12 are 30-day periods (baseline "months" 1-12) during which average monthly dose is stable (<10% variation) from the mean of 12 baseline months. M1-M7 are the seven 30-day periods ("months" 1-7) after the end of the baseline period. F1-F6 are six overlapping 60-day periods during which the average daily dose is computed for comparison to the baseline period (follow-up periods 1-6).

© 2019 Fenton JJ et al. *JAMA Network Open.*

eTable 1. Baseline Periods of Stable Opioid Use and Subsequent Tapering Events by Patient Characteristics and Year

Characteristic	Stable baseline periods, N*	Tapering event following stable baseline	
		n	%
**Total**	174,822	27,540	15.8
**Age category, y**			
18-34	4,694	790	16.8
35-49	36,568	5,826	15.9
50-64	90,970	14,396	15.8
>=65	42,590	6,528	15.3
**Sex**			
Male	83,195	12,459	15.0
Female	91,627	15,081	16.5
**Race/ethnicity**			
White	141,804	22,180	15.6
Black	18,393	3,030	16.5
Hispanic	8,794	1,428	16.2
Asian	1,333	199	14.9
Other/unknown	4,498	703	15.6
**Education**			
High school or less	79,592	12,245	15.4
More than high school	86,277	13,720	15.9
Unknown	8,953	1,575	17.6
**Urban vs. rural residence**			
Metropolitan	148,435	23,301	15.7
Micropolitan	15,033	2,453	16.3
Small town	7,307	1,133	15.5
Rural	3,785	588	15.5
Missing/unknown	262	65	24.8
**Comorbidity index**			
0	90,478	13,777	15.2
1	43,156	6,938	16.1
2	18,924	2,984	15.8
>=3	22,264	3,841	17.3
**Baseline opioid dose, MME**			
50-89	55,965	5,662	10.1
90-149	43,926	6,504	14.8
150-299	47,307	8,909	18.8
>=300	27,624	6,465	23.4
**Co-prescribed BDZ**			
No	125,871	19,222	15.3
Yes	48,951	8,318	17.0
**Recent drug overdose†**			
No	173,610	27,259	15.7
Yes	1,212	281	23.2
**Insurance status**			
Commercial	76,187	11,581	15.2
Medicare Advantage	98,635	15,959	16.2
Continued on next page			

© 2019 Fenton JJ et al. *JAMA Network Open.*

Year‡			
2008	9,254	1,057	11.4
2009	9,225	1,245	13.5
2010	11,339	1,555	13.7
2011	13,250	1,749	13.2
2012	17,784	2,489	14.0
2013	18,021	2,749	15.3
2014	18,846	2,823	15.0
2015	20,417	2,970	14.5
2016	28,219	4,741	16.8
2017	28,467	6,162	21.6

Abbreviations: MME=morphine milligram equivalents; BDZ=benzodiazepine

* There were 174,822 twelve-month baseline periods with opioid dose stability within 100,031 patients.

†Recent drug overdose defined as claims evidence of emergency or inpatient visit for all-drug overdose within 90 days of index opioid fill date

‡Year as of the first date of the follow-up period

© 2019 Fenton JJ et al. *JAMA Network Open.*

eTable 2. Poisson Regression Analyses Assessing Patient-Level Predictors of Tapering Based on a ≥30% Relative Dose Reduction Threshold (N=174,822 Stable Periods of Opioid Use Among 100,031 Patients)

Independent variable	IRR (95% CI)	*P* Value
**Age, y**		
18-34	Ref	
35-49	0.88 (0.80, 0.98)	0.015
50-64	0.84 (0.76, 0.92)	<.001
>=65	0.87 (0.79, 0.97)	0.009
**Sex**		
Male	Ref	
Female	1.15 (1.11, 1.19)	<.001
**Race/ethnicity**		
White	Ref	
Black	1.05 (1.00, 1.11)	0.054
Hispanic	1.01 (0.95, 1.09)	0.71
Asian	0.89 (0.74, 1.08)	0.22
Other/unknown	0.99 (0.90, 1.09)	0.86
**Education***		
High school or less	Ref	
More than high school	0.95 (0.92, 0.98)	<.001
Unknown	1.01 (0.94, 1.08)	0.85
**Urban vs. rural residence**		
Metro- or micropolitan or unknown	Ref	
Small town or Rural	1.03 (0.96, 1.09)	0.42
**Comorbidity index**		
0	Ref	
1	1.03 (0.99, 1.07)	0.15
2	1.03 (0.98, 1.09)	0.24
>=3	1.18 (1.13, 1.24)	<.001
**Baseline opioid dose, MME**		
50-89	Ref	
90-149	1.66 (1.58, 1.74)	<.001
150-299	2.10 (2.00, 2.19)	<.001
>=300	2.76 (2.62, 2.90)	<.001
**Co-prescribed BDZ**	1.02 (0.99, 1.06)	0.23
**Recent drug overdose†**	1.62 (1.41, 1.85)	0.001
**Insurance**		
Medicare Advantage	Ref	
Commercial	1.08 (1.04, 1.12)	<.001
**Year‡**		
Linear term per year (from 2008-2017)	1.061 (1.050, 1.071)	<.001
2016-2017	1.311 (1.248, 1.377)	<.001

Abbreviations: IRR=incidence rate ratio; CI=confidence interval; MME=morphine milligram equivalents; BDZ=benzodiazepine

* Median education of householders aged ≥25 years in same residential ZIP code

† Recent drug overdose defined as claims evidence of emergency or inpatient visit for all-drug overdose within 90 days of index opioid fill date

‡Study year was modeled by including a linear term for each increase in study year from 2008 to 2017 and an indicator variable for the 2016-2017 period.

© 2019 Fenton JJ et al. *JAMA Network Open.*

eTable 3. Complete Case Poisson Regression Analysis of Patient-Level Predictors of Long-Term Opioid Dose Tapering (N=162,345 Stable Periods of Opioid Use Among 90,613 Patients)

Independent variable	IRR (95% CI)	*P* Value
**Age, y**		
18-34	Ref	
35-49	0.88 (0.82, 0.94)	<.001
50-64	0.84 (0.78, 0.90)	<.001
>=65	0.86 (0.80, 0.93)	<.001
**Sex**		
Male	Ref	
Female	1.13 (1.10, 1.16)	<.001
**Race/ethnicity**		
White	Ref	
Black	1.05 (1.01, 1.09)	0.007
Hispanic	1.01 (0.96, 1.06)	0.78
Asian	0.93 (0.82, 1.07)	0.29
**Education***		
High school or less	Ref	
More than high school	0.99 (0.96, 1.01)	0.23
**Urban vs. rural residence**		
Metro- or micropolitan	Ref	
Small town or Rural	1.00 (0.95, 1.04)	0.87
**Comorbidity index**		
0	Ref	
1	1.04 (1.01, 1.07)	0.012
2	1.03 (0.99, 1.07)	0.20
>=3	1.13 (1.09, 1.18)	<.001
**Baseline opioid dose, MME**		
50-89	Ref	
90-149	1.47 (1.42, 1.53)	<.001
150-299	1.92 (1.86, 1.98)	<.001
>=300	2.52 (2.43, 2.61)	<.001
**Co-prescribed BDZ**	1.03 (1.00, 1.05)	0.05
**Recent drug overdose†**	1.37 (1.23, 1.52)	<.001
**Insurance**		
Medicare Advantage	Ref	
Commercial	1.08 (1.05, 1.11)	<.001
**Year‡**		
Linear term per year (from 2008-2017)	1.052 (1.045, 1.059)	<.001
2016-2017	1.209 (1.167, 1.252)	<.001

Abbreviations: IRR=incidence rate ratio; CI=confidence interval; MME=morphine milligram equivalents; BDZ=benzodiazepine

* Median education of householders aged ≥25 years in same residential ZIP code

† Recent drug overdose defined as claims evidence of emergency or inpatient visit for all-drug overdose within 90 days of index opioid fill date

‡Study year was modeled by including a linear term for each increase in study year from 2008 to 2017 and an indicator variable for the 2016-2017 period.

© 2019 Fenton JJ et al. *JAMA Network Open.*

eTable 4. Poisson Regression Analyses Assessing Patient-Level Predictors of Tapering, Restricted to Follow-up Periods in 2015-2017 (N=77,103 Stable Periods of Opioid Use Among 54,761 Patients)

**Independent variable**	**IRR (95% CI)**	***P* Value**
**Age, y**		
18-34	Ref	
35-49	1.02 (0.90, 1.15)	0.81
50-64	0.95 (0.85, 1.07)	0.42
>=65	0.96 (0.85, 1.08)	0.50
**Sex**		
Male	Ref	
Female	1.13 (1.10, 1.17)	<.001
**Race/ethnicity**		
White	Ref	
Black	1.05 (1.00, 1.10)	0.038
Hispanic	1.03 (0.97, 1.10)	0.30
Asian	0.96 (0.80, 1.16)	0.67
Other/unknown	0.96 (0.87, 1.05)	0.32
**Education**		
High school or less	Ref	
More than high school	1.00 (0.97, 1.04)	0.93
Unknown	1.13 (1.07, 1.19)	<.001
**Urban vs. rural residence**		
Metro- or micropolitan or unknown	Ref	
Small town or Rural	1.04 (0.98, 1.10)	0.20
**Comorbidity index**		
0	Ref	
1	1.06 (1.02, 1.10)	0.003
2	1.07 (1.02, 1.13)	0.005
>=3	1.18 (1.13, 1.23)	<.001
**Baseline opioid dose, MME**		
50-89	Ref	
90-149	1.61 (1.53, 1.68)	<.001
150-299	2.16 (2.07, 2.25)	<.001
>=300	2.95 (2.82, 3.10)	<.001
**Co-prescribed BDZ**	0.98 (0.95, 1.01)	0.23
**Recent drug overdose***	1.26 (1.09, 1.45)	0.001
**Insurance**		
Medicare Advantage	Ref	
Commercial	1.17 (1.13, 1.22)	<.001

Abbreviations: IRR=incidence rate ratio; CI=confidence interval; MME=morphine milligram equivalents; BDZ=benzodiazepine

* Recent drug overdose defined as claims evidence of emergency or inpatient visit for all-drug overdose within 90 days of index opioid fill date

© 2019 Fenton JJ et al. *JAMA Network Open.*

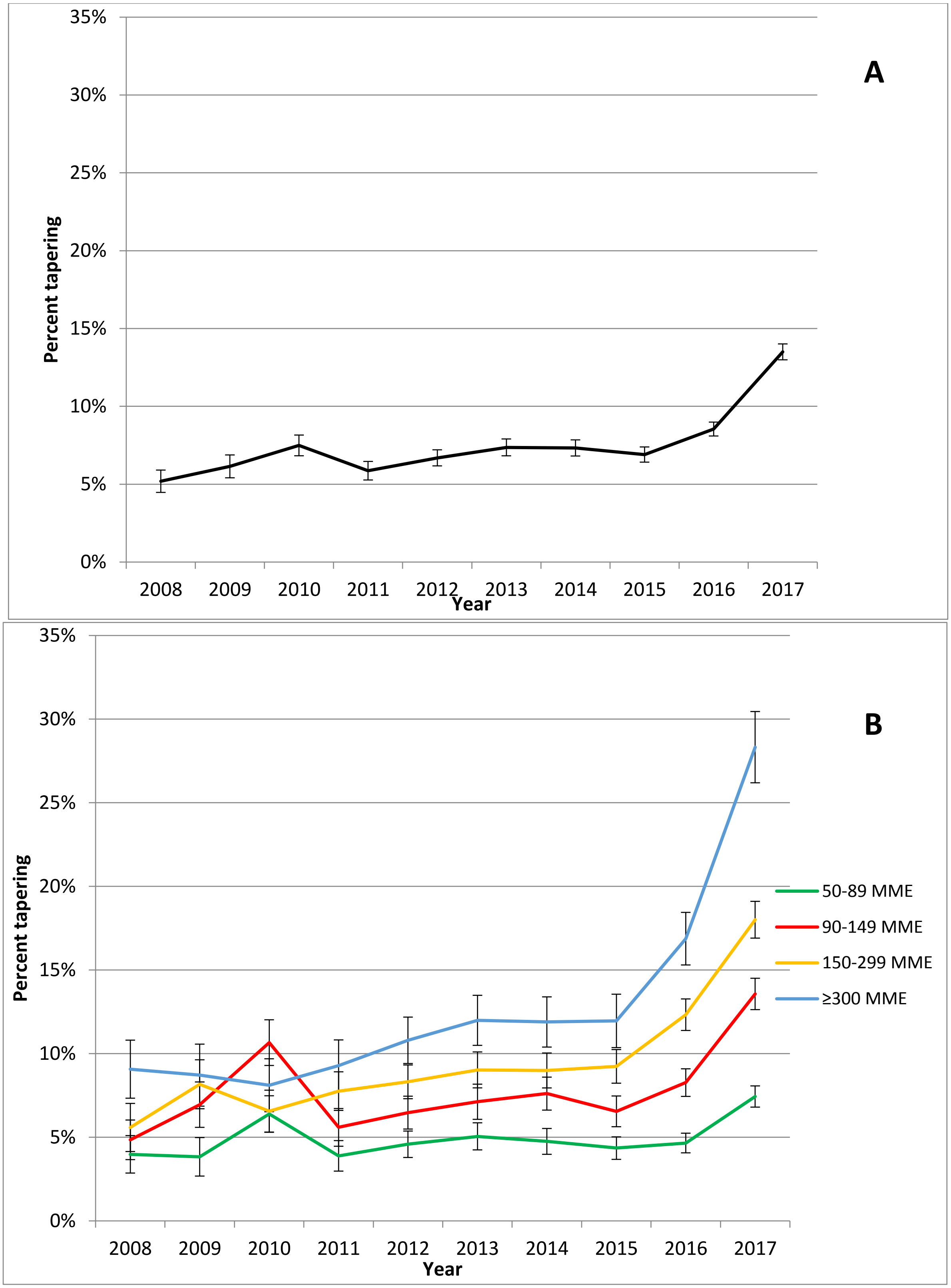

eFigure 2. Age- and Sex-Standardized Rates of Tapering Based on a ≥30% Relative Dose Reduction Threshold Among Patients Using Long-Term Opioid Therapy, 2008-2017. Panel A shows trends among the overall population and Panel B shows trends by baseline dosage in morphine milligram equivalents (MME). Yearly rates are age- and sex-standardized. Error bars give 95% confidence intervals.

© 2019 Fenton JJ et al. *JAMA Network Open.*

eTable 5. Beta Regression Analysis Assessing Patient-Level Predictors of Higher Maximum Tapering Velocity (N=27,540 Tapering Events Among 25,471 Patients)

Independent variable	OR* (95% CI)	*P* Value
**Age, y**		
18-34	Ref	
35-49	0.97 (0.92, 1.03)	0.36
50-64	0.96 (0.91, 1.13)	0.13
>=65	0.98 (0.93, 1.04)	0.57
**Sex**		
Male	Ref	
Female	1.01 (0.99, 1.02)	0.49
**Race/ethnicity**		
White	Ref	
Black	1.03 (1.00, 1.05)	0.078
Hispanic	0.99 (0.95, 1.02)	0.46
Asian	0.97 (0.89, 1.07)	0.55
Other/unknown	1.02 (0.96, 1.07)	0.57
**Education†**		
High school or less	Ref	
More than high school	0.96 (0.94, 0.97)	<.001
Unknown/missing	1.00 (0.97, 1.04)	0.88
**Urban vs. rural residence**		
Metro- or micropolitan or unknown	Ref	
Small town or Rural	1.02 (0.98, 1.05)	0.39
**Comorbidity index**		
0	Ref	
1	1.00 (0.98, 1.02)	0.92
2	1.03 (1.00, 1.06)	0.07
>=3	1.08 (1.05, 1.11)	<.001
**Baseline opioid dose, MME**		
50-89	Ref	
90-149	1.11 (1.09, 1.14)	<.001
150-299	1.12 (1.10, 1.15)	<.001
>=300	1.10 (1.08, 1.13)	<.001
**Co-prescribed BDZ**	1.01 (0.99, 1.03)	0.20
**Recent drug overdose‡**	1.20 (1.10, 1.32)	<.001
**Insurance**		
Medicare Advantage	Ref	
Commercial	0.99 (0.97, 1.01)	0.25
**Year§**		
Linear term per year (from 2008-2017)	0.98 (0.98, 0.99)	<.001
2016-2017	1.06 (1.04, 1.09)	<.001

Abbreviations: OR=odds ratio; CI=confidence interval; MME=morphine milligram equivalents; BDZ=benzodiazepine

*Odds ratios in the beta regression model estimate the change in the ratio V/(1-V) associated with the covariate relative to the referent, where V is the maximum dose reduction velocity. Hence odds ratios > 1.0 denote covariates associated with faster maximum dose reduction velocities.

† Median education of householders aged ≥25 years in same residential ZIP code

‡ Recent drug overdose defined as claims evidence of emergency or inpatient visit for all-drug overdose within 90 days of index opioid fill date

§Study year was modeled by including a linear term for each increase in study year from 2008 to 2017 and an indicator variable for the 2016-2017 period.

© 2019 Fenton JJ et al. *JAMA Network Open.*

eTable 6. Complete Case Linear Regression Analysis Assessing Patient-Level Predictors of Maximum Tapering Velocity (N=25,401 Tapering Events Among 23,400 Patients)

Characteristic	β* (95% CI)	*P* Value
**Age, y**		
18-34	Ref	
35-49	-0.5 (-1.8, 0.9)	0.49
50-64	-1.0 (-2.3, 0.4)	0.15
>=65	-0.1 (-1.5, 1.3)	0.87
**Sex**		
Male	Ref	
Female	0.1 (-0.4, 0.5)	0.80
**Race/ethnicity**		
White	Ref	
Black	0.6 (-0.1, 1.3)	0.09
Hispanic	0.0 (-1.0, 0.9)	0.93
Asian	-0.2 (-2.6, 2.1)	0.84
**Education†**		
High school or less	Ref	
More than high school	-1.2 (-1.6, -0.8)	<.001
**Urban vs. rural residence**		
Metro- or micropolitan	Ref	
Small town or Rural	0.2 (-0.7, 1.0)	0.73
**Comorbidity index**		
0	Ref	
1	-0.1 (-0.6, 0.4)	0.76
2	0.7 (0.0, 1.4)	0.056
>=3	1.7 (1.0, 2.4)	<.001
**Baseline opioid dose, MME**		
50-89	Ref	
90-149	2.6 (2.0, 3.2)	<.001
150-299	2.9 (2.3, 3.4)	<.001
>=300	2.5 (1.8, 3.1)	<.001
**Co-prescribed BDZ**	0.3 (-0.2, 0.8)	0.19
**Recent drug overdose‡**	4.8 (2.4, 7.1)	<.001
**Insurance**		
Medicare Advantage	Ref	
Commercial	-0.2 (-0.7, 0.3)	0.46
**Year§**		
Linear term per year (from 2008-2017)	-0.4 (-0.5, -0.3)	<.001
2016-2017	1.4 (0.7, 2.1)	<.001
**Constant**	25.3 (23.8, 26.8)	<.001

Abbreviations: BDZ=benzodiazepine, MME=Morphine milligram equivalents
*β coefficients estimate the percentage change maximum dose reduction velocity associated with covariates. Positive estimates indicate more rapid maximum velocity of dose reduction.
† Median education of householders aged ≥25 years in same residential ZIP code
‡ Recent drug overdose defined as claims evidence of emergency or inpatient visit for all-drug overdose within 90 days of index opioid fill date
§Study year was modeled by including a linear term for each increase in study year from 2008 to 2017 and an indicator variable for the 2016-2017 period.

© 2019 Fenton JJ et al. *JAMA Network Open.*

eTable 7. Logistic Regression Analysis of Patient-Level Predictors of Maximum Tapering Velocity Exceeding 40% per Month (N=27,540 Tapering Events Among 25,471 Patients)

**Independent variable**	**OR (95% CI)**	***P* Value**
**Age, y**		
18-34	Ref	
35-49	0.91 (0.75, 1.11)	0.34
50-64	0.85 (0.70, 1.03)	0.10
>=65	0.97 (0.80, 1.19)	0.80
**Sex**		
Male	Ref	
Female	1.01 (0.95, 1.08)	0.68
**Race/ethnicity**		
White	Ref	
Black	1.10 (1.00, 1.22)	0.053
Hispanic	1.04 (0.90, 1.20)	0.58
Asian	0.84 (0.57, 1.25)	0.39
Other/unknown	0.97 (0.80, 1.19)	0.80
**Education**		
High school or less	Ref	
More than high school	0.88 (0.82, 0.94)	<.001
Unknown/missing	1.02 (0.88, 1.17)	0.82
**Urban vs. rural residence**		
Metro- or micropolitan or unknown	Ref	
Small town or Rural	1.06 (0.94, 1.21)	0.35
**Comorbidity index**		
0	Ref	
1	1.03 (0.96, 1.12)	0.40
2	1.14 (1.03, 1.26)	0.014
>=3	1.34 (1.22, 1.47)	<.001
**Baseline opioid dose, MME**		
50-89	Ref	
90-149	1.48 (1.34, 1.63)	<.001
150-299	1.55 (1.41, 1.70)	<.001
>=300	1.38 (1.25, 1.53)	<.001
**Co-prescribed BDZ**	1.08 (1.01, 1.15)	0.032
**Recent drug overdose**	1.79 (1.37, 2.34)	<.001
**Insurance**		
Medicare Advantage	Ref	
Commercial	1.07 (1.00, 1.15)	0.067
**Year†**		
Linear term per year (from 2008-2017)	0.95 (0.93, 0.97)	<.001
2016-2017	1.17 (1.06, 1.30)	0.002

Abbreviations: OR=odds ratio; CI=confidence interval; MME=morphine milligram equivalents; BDZ=benzodiazepine

* Recent drug overdose defined as claims evidence of emergency or inpatient visit for all-drug overdose within 90 days of index opioid fill date

†Study year was modeled by including a linear term for each increase in study year from 2008 to 2017 and an indicator variable for the 2016-2017 period.

© 2019 Fenton JJ et al. *JAMA Network Open.*

eReference

1. Himstreet J, Popish S, Robeck I, Saenger M. Pain Management Opioid Taper Decision Tool. U.S. Washington, DC: Department of Veterans Affairs, 2016. (https://www.pbm.va.gov/AcademicDetailingService/Documents/Pain_Opioid_Taper_Tool_IB_10_939_P96820.pdf. Accessed January 28, 2019.
